# P02.130. The relationship of client expectations of massage to changes in pain and affect: results from a practice-based research study

**DOI:** 10.1186/1472-6882-12-S1-P186

**Published:** 2012-06-12

**Authors:** K Boulanger, S Campo

**Affiliations:** 1 University of Iowa, San Jose, USA; 2 Community and Behavioral Health, University of Iowa, Iowa City, USA

## Purpose

The purpose of this study was to examine whether client expectations of massage were related to changes in pain and affect after one massage therapy session.

## Methods

Practice-based research was used to collect client data (N=321) before and after a massage provided by one of 24 licensed massage therapists in Iowa. The pre-massage survey included items regarding their chief complaint, the client expectations of massage scale, the numeric rating scale for pain, and the Positive and Negative Affect Schedule Expanded form (PANAS-X). The post-massage survey included the same measures of pain and affect as well as demographics. Paired t-tests were used to test for significant changes in pain and affect. A structural equation model with outcome, interpersonal, clinical, and educational expectations as latent exogenous variables and changes in serenity, negative and positive affect, and pain as endogenous variables was tested using Mplus.

## Results

The mean age of the clients was 46 and 78% were female. Reasons for seeking massage were mainly musculoskeletal or for relaxation. There were significant improvements in negative affect, serenity, and pain pre-post massage, but not positive affect. Outcome expectations predicted changes in the serenity subscale of the PANAS-X and pain. Interpersonal expectations predicted changes in serenity. Clinical and educational expectations were not related to the outcomes used in this study.

## Conclusion

Client expectations of massage, specifically related to the benefits and interpersonal nature of massage therapy, are important constructs to measure to account for the changes in pain and affect. Although these findings are consistent with health behavior theories and empirical evidence, more studies with diverse client populations are needed.

